# Renoprotective Effects of MIT‐001 in Ischemia–Reperfusion Injury: Modulation of Ferroptosis, ROS and Fibrotic Markers

**DOI:** 10.1111/jcmm.70914

**Published:** 2025-10-22

**Authors:** Jin Ah Shin, Yunkyeong Hwang, Hyerim Park, Hyunsu Choi, Yoon‐Kyung Chang, Ki Ryang Na, Soon Ha Kim, Jae Young Kim, Young Rok Ham, Dae Eun Choi

**Affiliations:** ^1^ Department of Medical Science Chungnam National University Daejeon Republic of Korea; ^2^ Division of Nephrology Department of Internal Medicine Daejeon St Mary's Hospital, College of Medicine, The Catholic University of Korea Seoul Republic of Korea; ^3^ Clinical Research Institute Daejeon St Mary's Hospital Daejeon Republic of Korea; ^4^ Department of Nephrology Chungnam National University Hospital Daejeon Republic of Korea; ^5^ Mitoimmune Therapeutics Inc. Seoul Republic of Korea; ^6^ Hanmi Pharmaceutical Hwaseong‐si Republic of Korea

**Keywords:** chronic kidney disease, ferroptosis, ischemia–reperfusion injury, MIT‐001, oxidative stress, renal fibrosis

## Abstract

Renal ischemia–reperfusion injury (IRI) is a key driver of the progression from acute kidney injury (AKI) to chronic kidney disease (CKD), primarily through mechanisms involving oxidative stress, ferroptosis, and inflammation that promote fibrotic remodelling. This study investigates the therapeutic potential of MIT‐001, a mitochondria‐targeted reactive oxygen species (ROS) scavenger, in mitigating renal IRI. In vitro, MIT‐001 attenuated ferroptotic cell death and fibrotic responses in HK‐2 cells challenged with TGF‐β or RSL3. MIT‐001 restored GPX4 expression and activity, activated Nrf2 signalling, reduced lipid ROS and suppressed fibrogenic markers (α‐SMA, Snail, collagen IV), while preserving E‐cadherin levels. In a bilateral renal IRI mouse model, administration of MIT‐001 significantly improved renal function and histology. Oxidative stress (DHE staining), apoptosis (TUNEL) and ferroptosis (4‐HNE, xCT, GPX4) were markedly reduced. Additionally, MIT‐001 inhibited the NF‐κB/HMGB1 inflammatory axis and enhanced antioxidant defence via the Nrf2/HO‐1 pathway, resulting in decreased immune infiltration and fibrosis. These findings demonstrate that MIT‐001 confers renal protection by concurrently targeting oxidative stress, ferroptosis and inflammation, underscoring its promise as a therapeutic strategy to prevent AKI‐to‐CKD progression.

## 1 Introduction

Acute kidney injury (AKI) is increasingly recognised as a significant risk factor for the subsequent development of chronic kidney disease (CKD), a global health problem characterised primarily by progressive renal fibrosis [1]. Renal ischemia–reperfusion injury (IRI), a common cause of AKI, is a well‐established experimental model for investigating both the acute phase of kidney damage and its progression to CKD [2, 3]. The transition from AKI to CKD following renal IRI is driven by complex pathological mechanisms, including persistent oxidative stress, inflammation and ferroptosis [4].

Ferroptosis, a recently identified iron‐dependent form of regulated cell death, is characterised by excessive accumulation of lipid reactive oxygen species (ROS) due to impaired glutathione peroxidase 4 (GPX4) activity and the depletion of cellular glutathione (GSH) [5, 6]. Specifically, increased oxidative stress after renal IRI contributes to the activation of ferroptosis through GPX4 depletion, NF‐κB pathway activation, and enhanced lipid peroxidation, resulting in sustained inflammatory signalling and subsequent renal tissue damage [7, 8]. Moreover, lipid peroxidation products, such as 4‐hydroxynonenal (4‐HNE), exacerbate renal injury and fibrosis by further disrupting iron homeostasis and triggering ferroptotic cell death [9]. ROS also critically mediate the effects of transforming growth factor‐beta (TGF‐β), a key profibrotic cytokine responsible for promoting epithelial‐mesenchymal transition (EMT) and fibrogenesis in renal tissues [10, 11]. Hence, targeting oxidative stress and ferroptosis represents a promising therapeutic strategy for renal fibrosis‐associated with CKD.

MIT‐001 (NecroX‐7) is a mitochondria‐targeted antioxidant belonging to the cyclopentylamino carboxymethylthiazolylindole (NecroX) family. Previous studies have demonstrated that MIT‐001 effectively suppresses mitochondrial ROS overproduction, inhibits inflammatory cytokine expression, and restores mitochondrial function across various disease models [12, 13]. Specifically, MIT‐001 has been shown to exert protective effects against necrosis and inflammation in liver and kidney ischemia–reperfusion injury through the modulation of mitochondrial oxidative stress and inflammatory signalling pathways, including NF‐κB and MAPK [14, 15, 16]. Furthermore, MIT‐001 activates nuclear factor erythroid‐2‐related factor 2 (Nrf2), a central transcription factor regulating antioxidant responses, suggesting its potential efficacy in mitigating oxidative stress‐related renal injuries [17, 18]. Despite these promising findings, the specific effects and underlying mechanisms of MIT‐001 in ferroptosis regulation and fibrosis amelioration following renal IRI remain largely unexplored.

Recently, accumulating evidence has emphasised the significance of ferroptosis in renal fibrosis pathogenesis, suggesting therapeutic interventions targeting ferroptotic pathways may effectively prevent CKD progression. Studies using ferroptosis inhibitors or antioxidants, such as Formononetin, have demonstrated beneficial effects on renal fibrosis by enhancing Nrf2‐mediated antioxidant pathways and reducing ferroptotic damage in experimental CKD models [19, 20].

Therefore, the present study aimed to evaluate whether MIT‐001 ameliorates renal fibrosis induced by ischemia–reperfusion injury (IRI) by specifically targeting oxidative stress, inflammation and ferroptosis, thereby assessing its therapeutic potential in CKD‐associated renal fibrosis.

## 2 Results

### 2.1 MIT‐001 Reduces ROS, Ferroptosis and Inflammation Markers in TGF‐β‐Treated HK‐2 Cells

**FIGURE 1:**
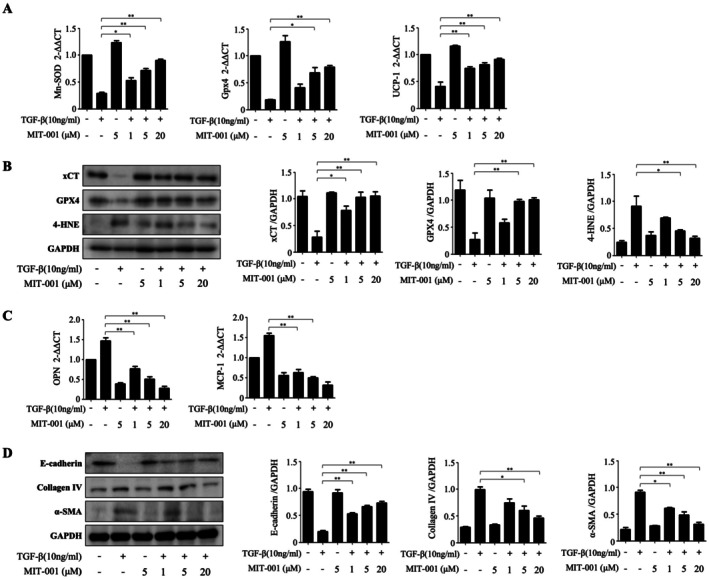
Effect of MIT‐001 on TGF‐β treated HK2 cells. (A) Expression of MnSOD, GPX4 and UCP1 mRNA in HK‐2 cells. (B) Representative Western blot results: xCT and GPX4 protein expression were decreased in HK‐2 cells after treatment with transgenic growth factor‐beta (TGF‐β). In these cells, the expression of xCT and GPX4 protein was gradually increased after treatment with various concentrations of MIT‐001. Protein and mRNA expression of 4‐HNE were increased in HK‐2 cells after TGF‐β treatment. (C) Osteopontin and MCP‐1 mRNA were overexpressed in HK‐2 cells treated with TGF‐β, then subsequently downregulated after MIT‐001 treatment. (D) Representative Western blot results: In HK‐2 cells treated with TGF‐β, E‐cadherin protein expression decreased and collagen IV and alpha smooth muscle actin (α‐SMA) protein expression increased. After those cells were subsequently treated with MIT‐001, E‐cadherin protein expression increased and collagen IV and α‐SMA protein expression decreased. *p < 0.01, **p < 0.001.

HK‐2 cells treated with TGF‐β (10 ng/mL) for 72 h followed by MIT‐001 (1, 5 or 20 μM) for 24 h showed concentration‐dependent increases in MnSOD, GPX4 and UCP1 mRNA levels compared to TGF‐β alone (Figure 1A). Western blot analyses revealed that TGF‐β decreased protein levels of xCT and GPX4, which were restored by MIT‐001 treatment. Additionally, TGF‐β increased 4‐HNE mRNA and protein expression, while MIT‐001 significantly reversed these increases (Figure 1B). TGF‐β treatment significantly elevated mRNA expression of inflammation‐related markers osteopontin (OPN) and MCP‐1, which were progressively reduced by MIT‐001 in a concentration‐dependent manner (Figure 1C). Furthermore, TGF‐β reduced protein levels of E‐cadherin and increased collagen IV, α‐SMA, Snail and Twist expression. MIT‐001 treatment reversed these effects, increasing E‐cadherin and decreasing fibrosis‐associated markers (Figure 1D).

### 2.2 MIT‐001 Inhibits Ferroptosis

**FIGURE 2:**
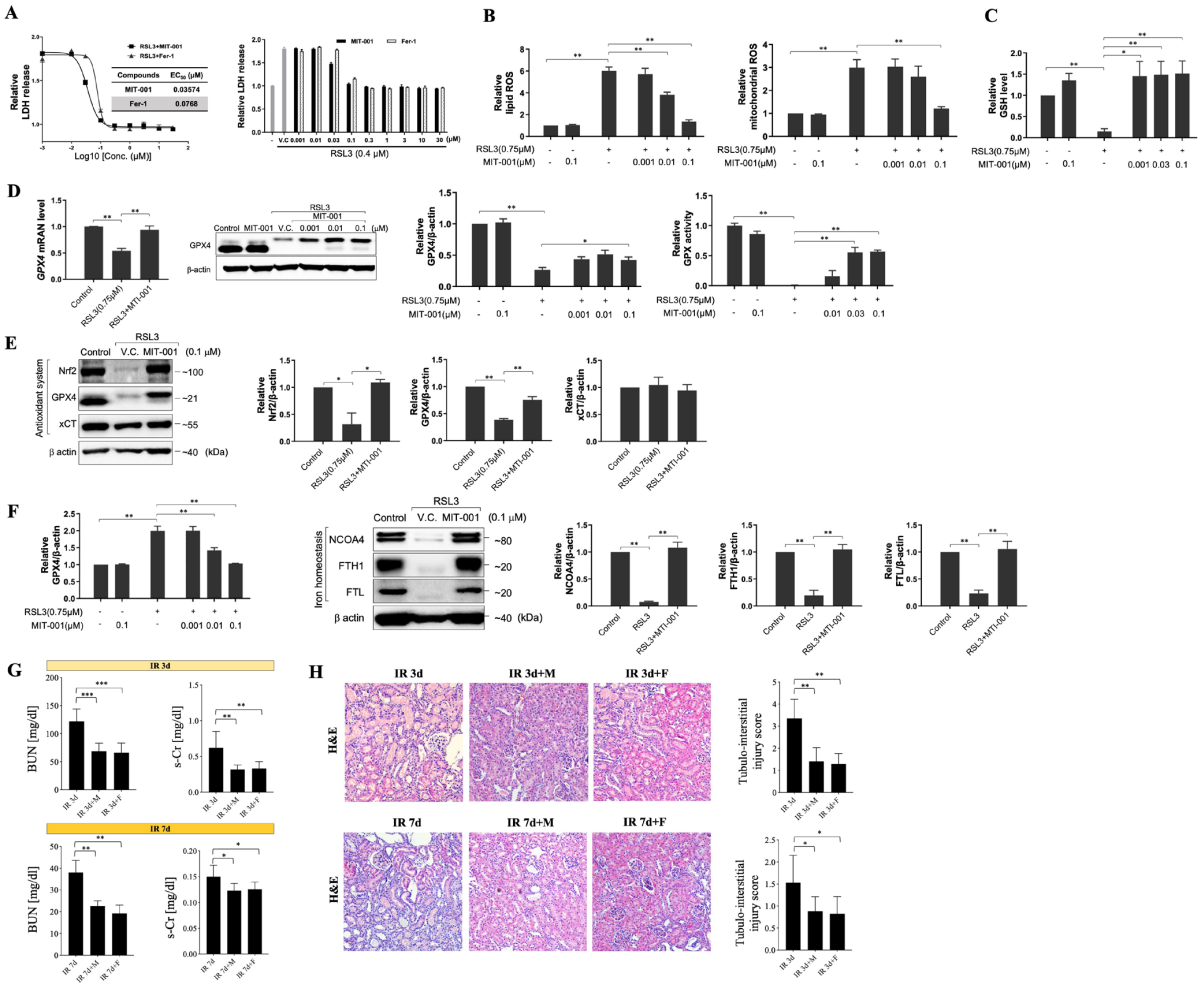
MIT‐001 Inhibits Ferroptosis. (A) In vitro comparative analysis of MIT‐001 and Fer‐1 on LDH inhibition after RSL3 (0.3 μM) treatment. EC50 values of MIT‐001 and Fer‐1 were decreased when RSL3 (0.3 μM) was treated together in HK‐2 cells. (B) After RSL3 (0.75 μM) treatment, MIT‐001 (0.001, 0.01 and 0.1 μM) reduced lipid ROS and mitochondrial ROS levels in HK‐2 cells. (C) GSH assay. Detection of GSH in HK‐2 cells administered RSL3 (0.3 μM) and treated with MIT‐001 (0.001, 0.01 and 0.1 μM). GSH levels recover upon treatment by concentration of MIT‐001. (D) MIT‐001 treatment group increases the mRNA level and protein expression level of GPX4 and upregulates the enzyme activity of GPX4 in HK‐2 cells. (E) MIT‐001 increases the protein expression of Nrf2 and GPX4, but the protein expression of xCT is not significantly different compared to RSL3‐only treatment in HK‐2 cells. (F) Iron homeostasis‐related protein level. MIT‐001 increases the protein expression of NCOA4, FTH1 and FTL in HK‐2 cells (0.001, 0.01 and 0.1 μM). (G) Renal function after renal ischemia–reperfusion injury (IRI) in mice. Blood urea nitrogen (BUN) and serum creatinine (s‐Cr) levels were significantly decreased in both IR 3d + M and IR 3d + F compared with IR 3d, and a similar pattern was observed at Day 7. (H) Representative kidney section with haematoxylin and eosin staining after renal IRI in mice. Dilated renal tubules, tubular necrosis, and inflammatory cell infiltration indicate tubulointerstitial damage at Days 3 and 7 after IRI, whereas treatment with MIT‐001 or ferrostatin‐1 significantly improved the tubulointerstitial damage score. 200× original magnification. Scale bar = 100 μm. IR 3d, untreated Day 3 renal IRI wild‐type mice (*n* = 7); IR 3d + M, Day 3 renal IRI mice treated with MIT‐001 (*n* = 7); IR 3d + F, Day 3 renal IRI mice treated with ferrostatin‐1 (*n* = 7); IR 7d, untreated Day 7 renal IRI mice (*n* = 7); IR 7d + M, Day 7 renal IRI mice treated with MIT‐001 (*n* = 7); IR 7d + F, Day 7 renal IRI mice treated with ferrostatin‐1 (*n* = 7). Bar charts show means ± standard deviation. Kruskal–Wallis *H* test, followed by a post hoc Bonferroni correction *p < 0.01, **p < 0.001, ***p < 0.001.

HK‐2 cells treated with the GPX4 inhibitor RSL3 (0.3 μM) exhibited increased cytotoxicity, as determined by lactate dehydrogenase (LDH) release. Co‐treatment with MIT‐001 significantly reduced LDH release, showing EC₅₀ values comparable to those of the ferroptosis inhibitor ferrostatin‐1 (Fer‐1) (Figure 2A). MIT‐001 also attenuated both cellular and mitochondrial lipid reactive oxygen species (ROS) levels following RSL3 (0.75 μM) exposure (Figure 2B). In addition, intracellular glutathione (GSH) levels were restored in a concentration‐dependent manner by MIT‐001 after RSL3 (0.3 μM) treatment (Figure 2C). RT‐PCR and Western blot analyses revealed that MIT‐001 upregulated GPX4 mRNA and protein expression, as well as GPX4 enzymatic activity, compared to cells treated with RSL3 alone. MIT‐001 also increased Nrf2 protein levels, while xCT expression remained unchanged (Figure 2D). Furthermore, Western blot analysis showed that MIT‐001 enhanced the expression of iron homeostasis‐related proteins, including NCOA4, FTH1 and FTL, in comparison to the RSL3‐only treated group (Figure 2F). In an in vivo study, the effects of MIT‐001 and ferrostatin‐1 were evaluated in mice following renal IRI. Renal function, assessed by blood urea nitrogen (BUN) and serum creatinine (s‐Cr), was significantly improved in MIT‐001–treated mice (IR 3d + M and IR 7d + M) as well as in ferrostatin‐1–treated mice (IR 3d + F and IR 7d + F), compared with untreated IRI mice at Days 3 and 7 post‐IRI (Figure 2G). Histological evaluation (H&E staining) revealed severe tubular injury and inflammatory infiltration in untreated IRI kidneys, whereas both MIT‐001‐treated and ferrostatin‐1‐treated groups exhibited notably reduced tissue injury scores. No significant differences were observed between the MIT‐001‐treated and ferrostatin‐1‐treated groups (Figure 2H).

### 2.3 MIT‐001 Improves Renal Function and Renal Tissue Injuries in IRI Mice

**FIGURE 3:**
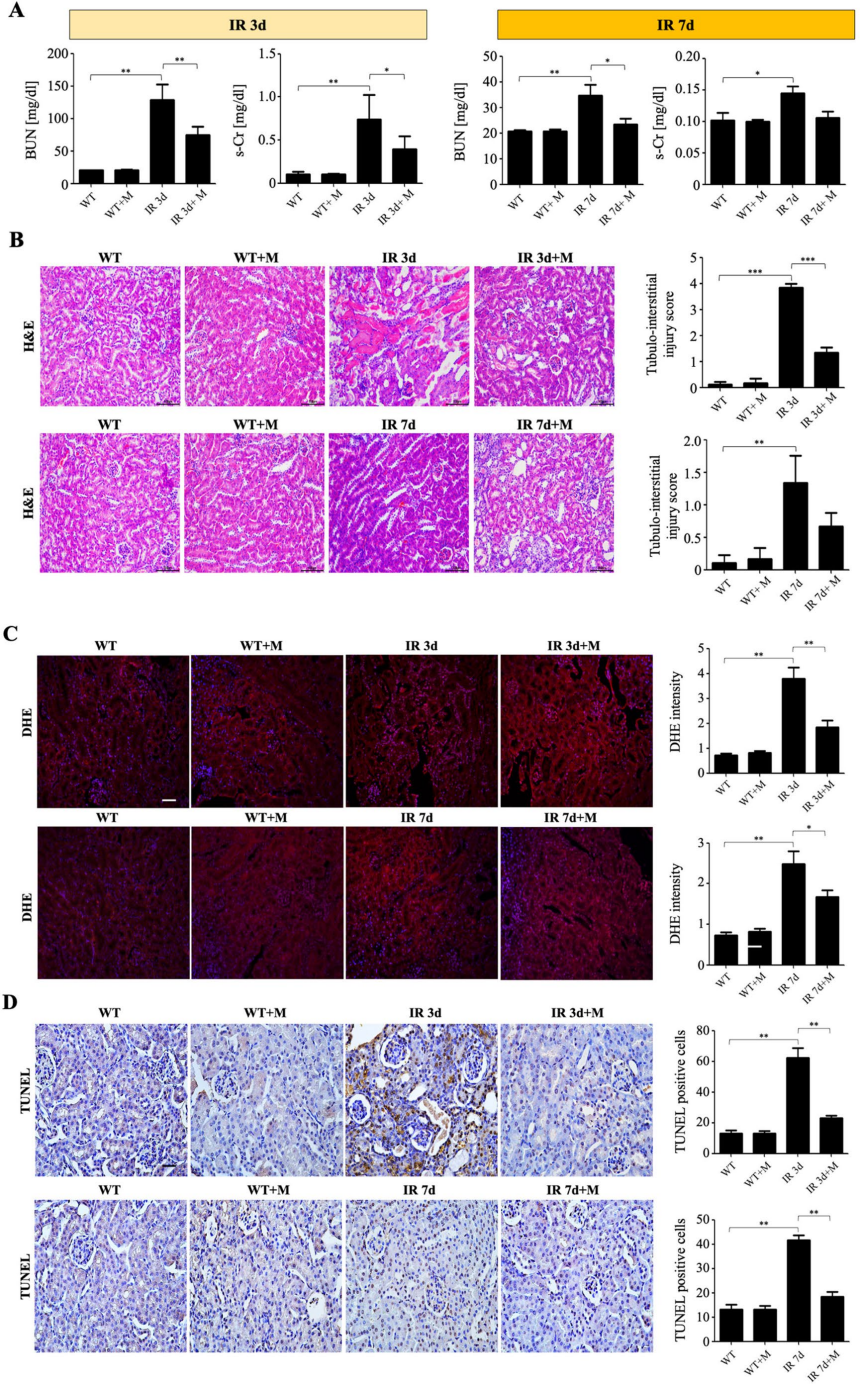
Renal function and histology after ischemia–reperfusion injury (IRI) in wild‐type mice. (A) Blood urea nitrogen and serum creatinine (s‐Cr) levels were significantly decreased in both IR 3d + M and IR 7d + M mice. (B) Typical kidney sections with haematoxylin and eosin staining. Dilated renal tubules, tubular necrosis, and inflammatory cell infiltration indicate tubulointerstitial damage. 200× original magnification. Scale bar = 100 μm. (C) Representative micrographs of dihydroethidium (DHE) staining in renal sections, quantified by fluorescence intensity. 200× original magnification. Scale bar = 50 μm. (D) Representative TUNEL‐stained renal sections. 400× original magnification. Scale bar = 200 μm. WT, wild‐type mice, sham treatment (*n* = 5 for 3d, *n* = 5 for 7d); WT + M, wild‐type mice treated with MIT‐001 (*n* = 7 for 3d, *n* = 7 for 7d); IR 3d, untreated Day 3 renal IRI wild‐type mice (*n* = 10); IR 3d + M, Day 3 renal IRI mice treated with MIT‐001 (*n* = 10); IR 7d, untreated Day 7 renal IRI mice (*n* = 10); IR 7d + M, Day 7 renal IRI mice treated with MIT‐001 (*n* = 10) (0.001, 0.01 and 0.1 μM). Bar charts show means ± standard deviation. Kruskal–Wallis *H* test, followed by a post hoc Bonferroni correction *p < 0.01, **p < 0.001.

Renal function, assessed by blood urea nitrogen and serum creatinine, was significantly improved in MIT‐001‐treated mice (IR 3d + M and IR 7d + M) compared with untreated IRI mice at Days 3 and 7 post‐IRI (Figure 3A). Histological evaluation (H&E staining) showed severe tubular injury and inflammatory infiltration in untreated IRI kidneys. MIT‐001‐treated groups exhibited notably reduced tissue injury scores (Figure 3B). DHE staining demonstrated significantly lower ROS production in kidney tissues from MIT‐001‐treated groups (IR 3d + M and IR 7d + M) compared to untreated IRI groups (IR 3d, IR 7d) (Figure 3C). TUNEL assay confirmed fewer apoptotic cells in kidneys from MIT‐001‐treated mice (IR 3d + M, IR 7d + M) relative to untreated groups (Figure 3D).

### 2.4 MIT‐001 Suppresses Ferroptosis in IRI Kidneys

**FIGURE 4:**
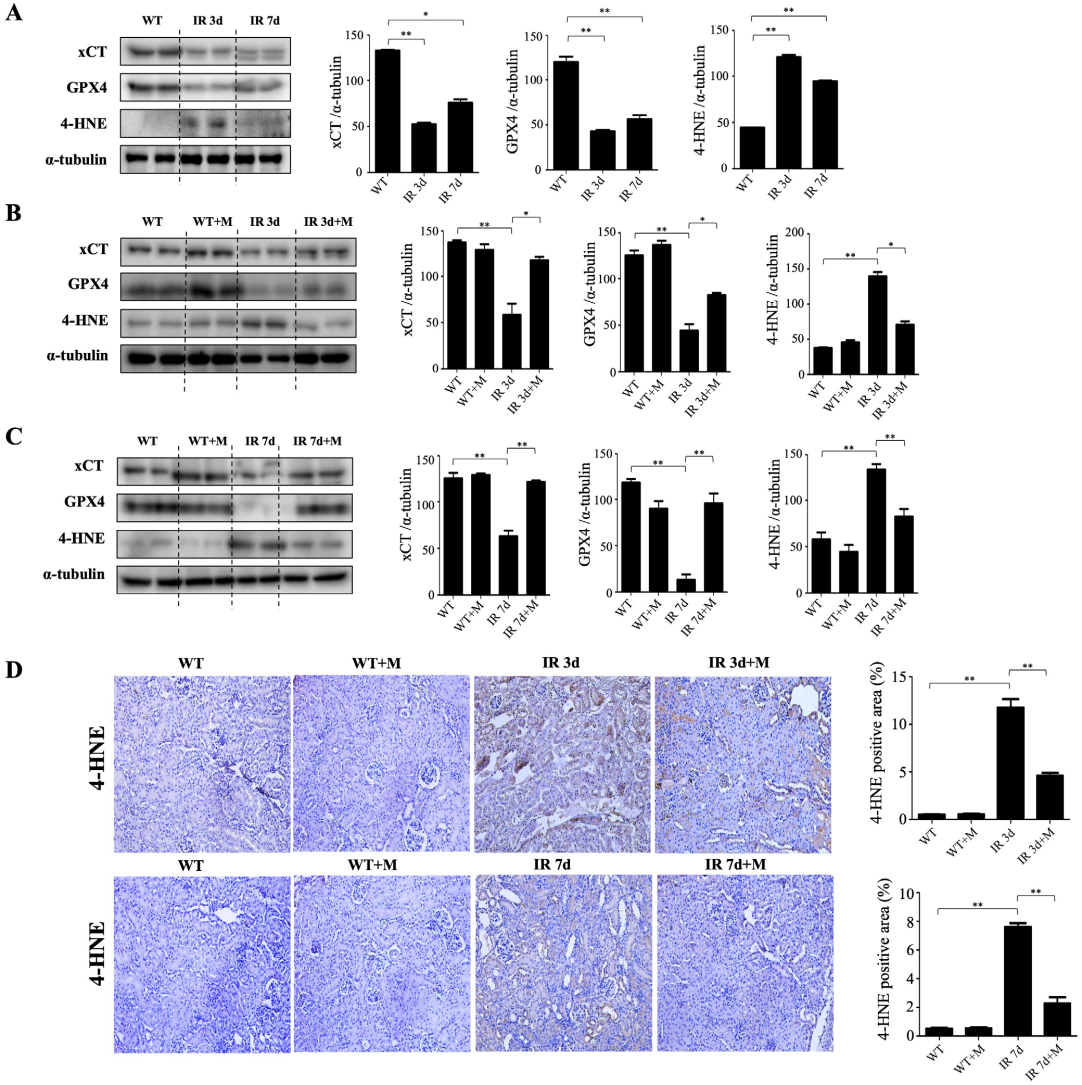
Anti‐ferroptosis effect of MIT‐001 in the kidneys of mice with ischemia–reperfusion injury (IRI). (A) Representative Western blot results for renal lysis: XCT and *GPX4* protein expression decreased and 4‐HNE protein expression increased in the kidneys of IR 3d and IR 7d mice compared with the kidneys of wild‐type (WT) mice. (B) Representative Western blot results for renal lysis: XCT and *GPX4* protein expression increased and 4‐HNE protein expression decreased in the kidneys of IR 3d + M mice compared with the kidneys of IR 3d mice. (C) Representative Western blot results for renal lysis: XCT and GPX4 protein expression increased and 4‐HNE protein expression decreased in the kidneys of IR 7d + M mice compared with the kidneys of IR 7d mice. (D) Representative Immunohistochemical kidney sections. Immunohistochemical staining with lipid peroxidation marker 4‐HNE showed a significant decrease in 4‐HNE expression in the kidneys of IR 3d + M and IR 7d + M mice. 200× original magnification. Scale bar = 50 μm. Bar charts show means ± standard deviation (whiskers). WT, wild‐type mice, sham treatment; WT + M, wild‐type mice treated with MIT‐001; IR 3d, untreated Day 3 renal IRI wild‐type mice; IR 3d + M, Day 3 renal IRI mice treated with MIT‐001; IR 7d, untreated Day 7 renal IRI mice; IR 7d + M, Day 7 renal IRI mice treated with MIT‐001. *p < 0.01, **p < 0.001.

Western blot analysis indicated that MIT‐001 administration significantly restored protein levels of xCT and GPX4, which were downregulated in untreated IRI kidneys. Concurrently, increased 4‐HNE protein expression in untreated IRI groups was markedly reduced by MIT‐001 treatment (Figure 4A–C). Immunohistochemistry for 4‐HNE confirmed reduced lipid peroxidation in MIT‐001‐treated groups compared to untreated IRI mice (Figure 4D).

### 2.5 MIT‐001 Reduces Inflammation in IRI Kidneys

**FIGURE 5:**
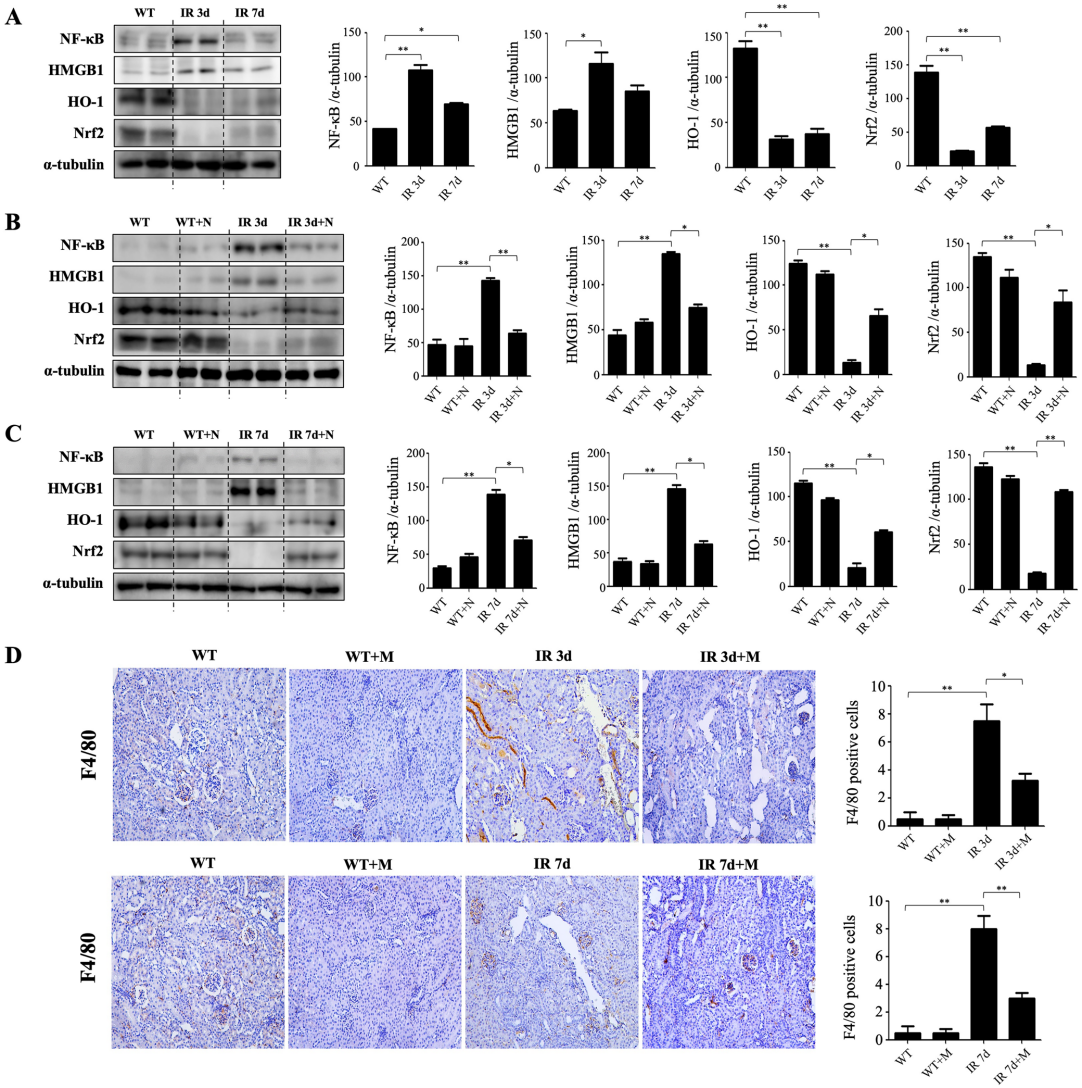
MIT‐001 protects against ischemia–reperfusion injury (IRI)–induced reactive inflammation in the kidney. (A–C) Representative Western blot results for kidney lysis: NF‐κB and HMGB1 expression decreased, and HO‐1 and Nrf2 expression increased in the kidneys of IR 3d + M and IR 7d + M mice compared with the kidneys of IR 3d and IR 7d mice. There was no significant difference in the kidneys of WT and WT + M mice. (D) Representative immunohistochemical kidney sections. Immunohistochemical staining using the macrophage marker F4/80 showed a significant decrease in F4/80 expression in the kidneys of IR 3d + M and IR 7d + M mice. 400× original magnification. Scale bar = 200 μm. WT, wild‐type mice, sham treatment (*n* = 5 for 3d, *n* = 5 for 7d); WT + M, wild‐type mice treated with MIT‐001 (*n* = 7 for 3d, *n* = 7 for 7d); IR 3d, untreated Day 3 renal IRI wild‐type mice (*n* = 10); IR 3d + M, Day 3 renal IRI mice treated with MIT‐001 (*n* = 10); IR 7d, untreated Day 7 renal IRI mice (*n* = 10); IR 7d + M, Day 7 renal IRI mice treated with MIT‐001 (*n* = 10). Bar charts show means ± standard deviation. Kruskal–Wallis H test, followed by a post hoc Bonferroni correction *p < 0.01, **p < 0.001.

Western blot analyses showed elevated NF‐κB and HMGB1 expression in untreated IRI kidneys, which was notably decreased by MIT‐001 treatment. Conversely, HO‐1 and Nrf2 expression, reduced in untreated IRI kidneys, was increased in MIT‐001‐treated groups (Figure 5A–C). Immunohistochemistry for the macrophage marker F4/80 revealed significantly lower macrophage infiltration in MIT‐001‐treated kidneys compared with untreated groups (Figure 5D).

### 2.6 MIT‐001 Attenuates Renal Fibrosis Induced by IRI

**FIGURE 6:**
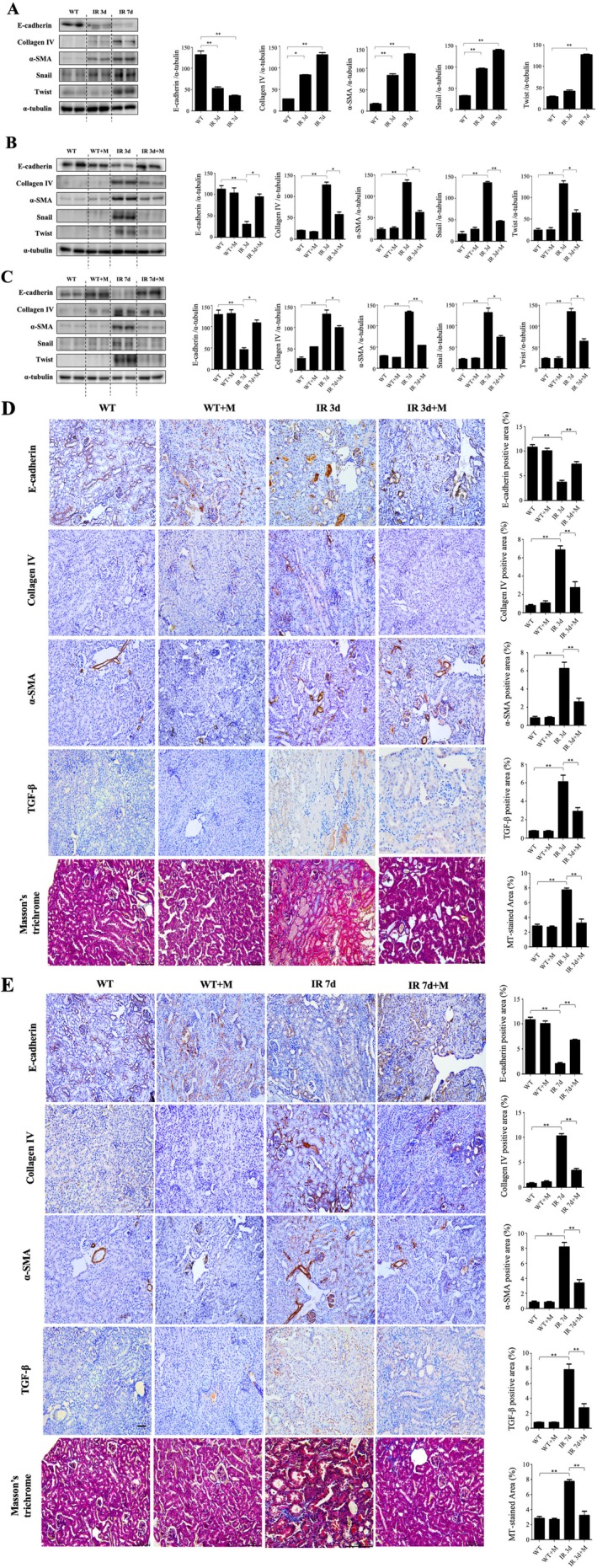
Effect of MIT‐001 on renal fibrosis. (A) Representative Western blot results for renal lysis: E‐cadherin protein expression decreased and collagen IV, α‐SMA, Snail and Twist protein expression increased in the kidneys of IR 3d and IR 7d mice compared with the kidneys of WT mice. (B) Representative Western blot results for renal lysis: E‐cadherin expression increased and collagen IV, α‐SMA, Snail and Twist protein expression decreased in the kidneys of IR 3d + M mice compared with the kidneys of IR 3d mice. (C) Representative Western blot results for renal lysis: E‐cadherin protein expression increased and collagen IV, α‐SMA, Snail and Twist protein expression decreased in the kidneys of IR 7d + M mice compared with the kidneys of IR 7d mice. (D, E) Representative immunohistochemical staining of kidney sections of IR 3d and IR 7d respectively. Staining was performed using cell–cell conjugated protein for epithelial cell marker E‐cadherin, epithelial and endothelial cell marker collagen IV, myofibroblast marker α‐SMA, and multiple cytokine TGF‐β. Representative photomicrographs of kidney sections stained with Masson trichrome are also presented. 200× original magnification. Scale bar = 100 μm. WT, wild‐type mice, sham treatment (*n* = 5 for 3d, *n* = 5 for 7d); WT + M, wild‐type mice treated with MIT‐001 (*n* = 7 for 3d, *n* = 7 for 7d); IR 3d, untreated Day 3 renal IRI wild‐type mice (*n* = 10); IR 3d + M, Day 3 renal IRI mice treated with MIT‐001 (*n* = 10); IR 7d, untreated Day 7 renal IRI mice (*n* = 10); IR 7d + M, Day 7 renal IRI mice treated with MIT‐001 (*n* = 10). Bar charts show means ± standard deviation. Kruskal‐Wallis H test, followed by a post hoc Bonferroni correction *p < 0.01; **p < 0.001.

Western blot results demonstrated significant decreases in E‐cadherin and increases in fibrosis markers (collagen IV, α‐SMA, Snail, Twist) in untreated IRI groups. MIT‐001 reversed these changes, increasing E‐cadherin and reducing fibrotic markers (Figure 6A–C). Immunohistochemical staining and Masson's trichrome staining further confirmed reduced fibrosis and increased E‐cadherin expression in MIT‐001‐treated kidneys compared to untreated IRI kidneys (Figure 6D,E).

## 3 Discussion

**FIGURE 7:**
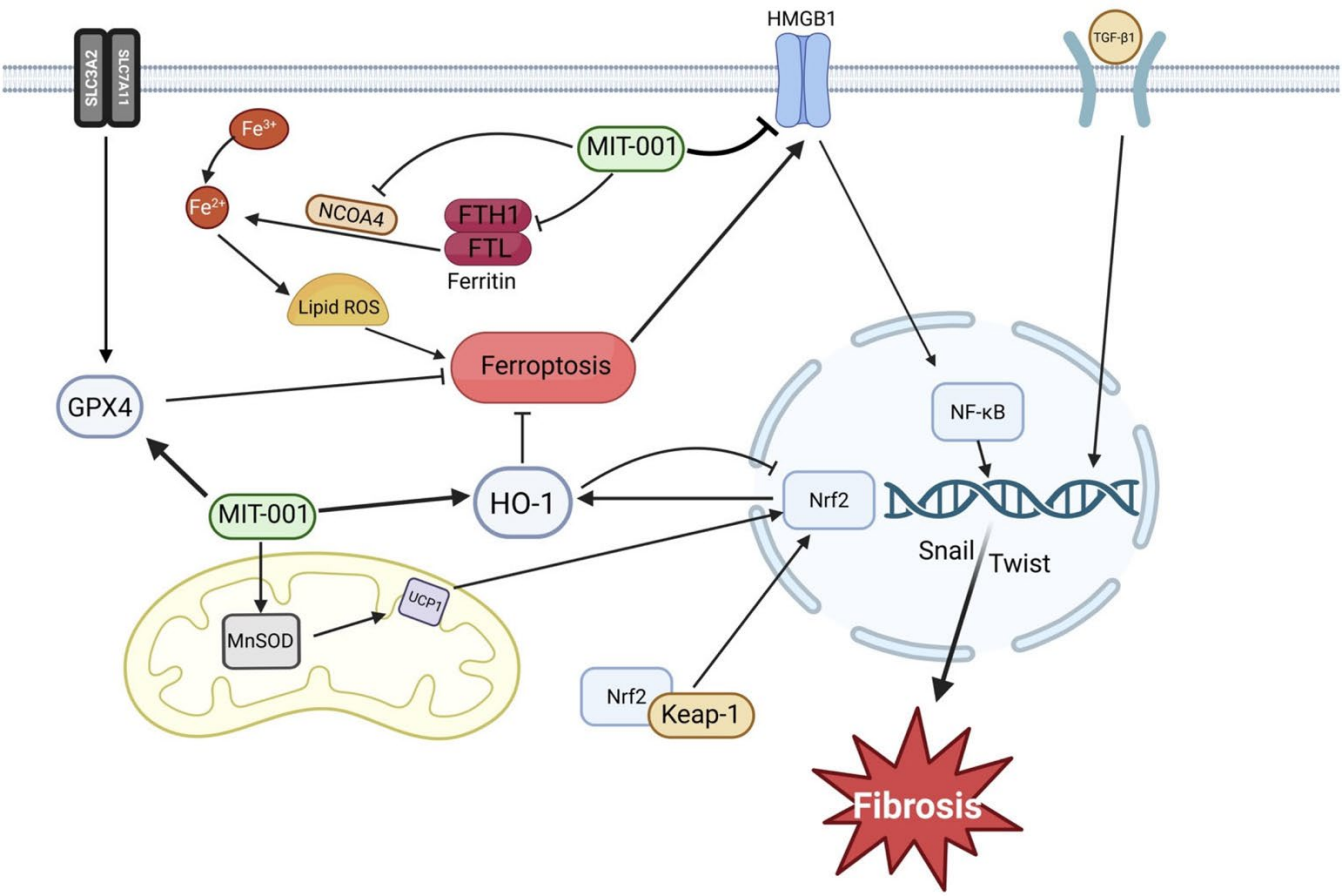
A schematic diagram showing how renal IRI increases renal injury and how MIT‐001 attenuates the resulting fibrosis. In IRI mice, increased lipid ROS induces ferroptosis, and HMGB1/NF‐κB signalling increases inflammation, contributing to renal fibrosis. However, MIT‐001 can activate MnSOD, UPC1, Nrf2, HO‐1 and Gpx4 and inhibit NCOA4, FTHL1, FTL and HMGB1/NF‐κB signalling, thereby improving IRI‐induced ferroptosis and fibrosis.

In this study, we provide robust evidence that MIT‐001, a mitochondrial‐targeted reactive oxygen species (ROS) scavenger, ameliorates renal fibrosis in IRI by simultaneously mitigating oxidative stress, inhibiting ferroptosis, suppressing inflammation, and disrupting epithelial‐mesenchymal transition (EMT) (Figure 7).

IRI exacerbates lipid peroxidation and ROS accumulation, promoting ferroptosis—a regulated form of cell death tightly linked to impaired iron metabolism and membrane lipid oxidation [5, 21]. In our model, 4‐HNE accumulation, decreased GPX4 and suppressed xCT expression confirmed ferroptotic activity post‐IRI. Treatment with MIT‐001 significantly reversed these markers, upregulating antioxidant systems such as GPX4, MnSOD and UCP1—key regulators of cellular redox homeostasis [22, 23, 24]. These observations align with previous reports identifying GPX4 suppression and lipid peroxidation as central events in renal tubular injury [25, 26].

The restoration of the GSH/GPX4 axis by MIT‐001 was especially significant given the importance of glutathione in controlling ferroptosis [9]. Our findings expand on prior studies showing the therapeutic benefits of ferroptosis inhibitors like Fer‐1 and NecroX compounds in CKD models [27, 28]. Notably, MIT‐001 demonstrated comparable potency to Fer‐1 in inhibiting RSL3‐induced ferroptosis, positioning it as a competitive candidate in the emerging class of ferroptosis‐targeted renal therapeutics. In addition, our new in vivo comparison with ferrostatin‐1 revealed that both MIT‐001– and ferrostatin‐1–treated mice showed significantly improved renal function (BUN, s‐Cr) and reduced histological injury scores compared with untreated IRI mice, with no significant differences between the two treatment groups. These findings highlight that MIT‐001 confers renoprotective effects comparable to an established ferroptosis inhibitor, thereby enhancing the translational relevance of our study.

MIT‐001 also showed substantial anti‐inflammatory activity by downregulating HMGB1 and NF‐κB while concurrently upregulating the Nrf2/HO‐1 signalling axis. HMGB1‐mediated NF‐κB activation is known to perpetuate inflammatory and fibrotic signalling [29], whereas activation of Nrf2/HO‐1 confers antioxidant protection and has been shown to reverse CKD‐related inflammation [19, 30]. The dual action of MIT‐001 in inhibiting the HMGB1/NF‐κB pathway and activating Nrf2/HO‐1 was consistent with previous findings in ageing models and reinforces its potential to interrupt the inflammatory–oxidative feedback loop central to CKD pathogenesis [17, 18] Inhibition of EMT, a critical process in renal fibrogenesis, was another key effect of MIT‐001. Renal fibrosis is marked by decreased E‐cadherin and increased mesenchymal markers such as α‐SMA, Snail and Twist—hallmarks of EMT driven by TGF‐β signalling [11, 31]. Our in vivo and in vitro results clearly demonstrated that MIT‐001 restored E‐cadherin levels and suppressed EMT‐related protein expression, suggesting that it effectively halts fibrotic transdifferentiation. This is in line with findings from other models where TGF‐β inhibition reduces renal fibrosis [20, 32].

Moreover, our use of DHE and TUNEL staining confirmed the reduction of ROS and apoptosis, respectively, in IRI kidneys treated with MIT‐001. These data suggest that the compound's anti‐ROS properties translate into protection against both ferroptotic and apoptotic cell death, thereby preserving renal architecture and function. This was further corroborated by reduced blood urea nitrogen and serum creatinine levels—established clinical markers of renal function.

Importantly, although MIT‐001 modulates several molecular pathways associated with IRI‐induced damage—including restoration of GPX4, activation of Nrf2/HO‐1, inhibition of NF‐κB/HMGB1 and reversal of EMT—it remains unclear whether these effects are the result of direct interactions with these molecular targets or occur secondarily via its established actions as a mitochondria‐targeted antioxidant. Current evidence, including our observations and previous reports on NecroX‐class compounds, suggests that the principal effect of MIT‐001 is the suppression of mitochondrial ROS, which leads to downstream modulation of ferroptosis, inflammatory signalling and fibrotic remodelling. Therefore, it is most plausible that the observed changes in pathway markers are secondary to the reduction of mitochondrial ROS, rather than the result of direct engagement with individual proteins such as GPX4 or Nrf2. Direct biochemical evidence for target selectivity—such as binding affinity studies, knockdown/knockout models, or functional rescue assays—was not performed in this study. Future mechanistic studies using genetic or proteomic profiling, or direct target engagement assays, will be needed to clarify whether MIT‐001 directly interacts with these pathways or exerts its pleiotropic effects solely by attenuating mitochondrial oxidative stress. In this regard, the therapeutic promise of MIT‐001 should be interpreted in the context of its primary ROS‐scavenging activity and the resulting broad‐spectrum secondary effects on cell death and inflammation, rather than as a highly selective modulator of individual molecular pathways.

A limitation of this study is that renal outcomes were only assessed at early time points (Days 3 and 7) following IRI. While these findings provide important mechanistic insights into the protective effects of MIT‐001, they do not capture the long‐term trajectory of AKI‐to‐CKD progression. Future studies with longer follow‐up periods (e.g., 4–6 weeks) will be necessary to more conclusively determine whether MIT‐001 can prevent the transition from acute to chronic kidney injury.

In conclusion, our findings underscore the therapeutic promise of MIT‐001 in IRI‐induced renal fibrosis. Through robust inhibition of ROS, suppression of ferroptosis, reversal of EMT and attenuation of inflammatory signalling, MIT‐001 addresses multiple pathological mechanisms contributing to CKD progression. These results not only clarify the molecular landscape of IRI‐associated renal fibrosis but also pave the way for further development of MIT‐001 as a multi‐targeted intervention in CKD.

## 4 Materials and Methods

### 4.1 Cell Culture and Drug Treatment

Immortalised HK‐2 cells were incubated in Dulbecco modified Eagle medium F12 (DMEM/F12: Welgene, Gyeongsan‐si, ROK) containing 10% fetal bovine serum (Life Technologies Inc., Gaithersburg, MD, USA) and 1% antibiotic–antimycotic at 37°C under 5% CO_2_. HK‐2 cells were plated and adhered (16–18 h) and then exposed to 10 ng/mL TGF‐β (R&D Systems, Minneapolis, MN, USA) for 72 h. Exposed cells were subsequently treated with 1, 5, or 20 μM MIT‐001 (MitoImmune Therapeutics Inc., Seoul, ROK) for 24 h. For RSL3‐induced ferroptosis assay, HK‐2 cells were pre‐treated with the indicated concentration of MIT‐001 for 20 min and then exposed to 0.3 or 0.75 μM RSL3 (Selleckchem, Huston, TX, USA) for 24 h.

### 4.2 LDH Assay

For cytotoxicity LDH assay, LDH assay kit (Dojindo, Mashiki‐machi, Kumamoto, Japan) was used according to the manufacturer's protocol. Briefly, 5 × 10^3^ HK‐2 cells were seeded on the 96‐well culture plate and incubated for 16–18 h and then treated with 0.001, 0.01, 0.03, 0.1, 0.3, 1, 3, 10 and 30 μM MIT‐001 for 20 min. Subsequently, cells were exposed to 0.3 μM RSL3 for 24 h. After RSL3 treatment, supernatants of cell culture media were transferred to a new 96‐well plate and the working solution of the assay kit was added to those supernatants. The mixtures were incubated for 20 min in the dark. To complete the assay, the stop solution was added to those mixtures. The LDH release was determined by absorbance at 490 nm using the SpectraMax iD3 multi‐mode microplate reader (Molecular Devices, San Jose, CA, USA).

### 4.3 FACS Analysis

Cellular lipid ROS and mitochondrial lipid ROS were measured by BODIPY 581/591 C11 (Invitrogen, Waltham, MA, USA) and MitoPerOx (Abcam, Cambridge, UK), respectively, fluorescent staining and using the BD FACSLyricTM instrument (BD Bioscience, Becton Dickinson, Franklin Lakes, NJ, USA). Briefly, 2.5 × 10^5^ HK‐2 cells were stained with fluorescent dyes for 20 min and then washed with phosphate‐buffered saline (PBS). The washed cells were resuspended in PBS and subjected to FACS analysis. FACS analysis was performed according to the fluorescent dye manufacturer's protocol. Relative lipid ROS or mitochondrial lipid ROS were presented as relative values to control.

### 4.4 GSH Level Measurement

The cellular GSH level was determined using the GSH assay kit (Abcam, Cambridge, UK) according to the manufacturer's protocol. Briefly, 5 × 10^5^ HK‐2 cells were homogenised in 0.1 mL of 5% sulfosalicylic acid solution and then centrifuged at 12,000 g at 4°C for 20 min. Supernatants were collected and diluted with assay buffer. The diluted samples were plated and mixed with enzyme buffer. The detection of the GSH level was performed with the SpectraMax iD3 multi‐mode microplate reader by kinetic measurement of sample absorbance at 450 nm. Relative GSH level was presented as relative values to control.

### 4.5 GPX Activity Measurement

The activity of glutathione peroxidases (GPX) was determined using the Glutathione Peroxidase Assay Kit (Colorimetric) (Abcam, Cambridge, UK), according to the manufacturer's protocol. Briefly, 5 × 10^5^ HK‐2 cells were homogenised in 0.2 mL ice‐cold assay buffer, and 50 μL of enzyme extract was taken for the analysis. Activity determination was performed with the SpectraMax iD3 multi‐mode microplate reader by measuring sample absorbance at 340 nm. Relative GPX activity was presented as relative values to control.

### 4.6 Animal Model and Ethical Approval

All animal experiments were approved by the Animal Use and Care Committee of Chungnam National University School of Medicine (approval number: 202012A‐CNU‐159) and conducted in accordance with institutional guidelines for the ethical use of animals. Male C57BL/6 mice (8 weeks old) were obtained from Samtako Bio Korea (Osan, Gyeonggi, ROK) and housed under controlled environmental conditions (12 h light/dark cycle) with free access to food and water. Mice were randomly assigned to 3 day and 7 day experimental groups, each consisting of four subgroups: WT, WT + MIT‐001, IR and IR + MIT‐001. Each subgroup included 5 to 10 mice (Figure S2). Renal ischemia–reperfusion injury (IRI) was induced under ketamine anaesthesia (60 mg/kg, intraperitoneally) by bilateral clamping of the renal pedicles for 25 min, following the protocol described by Versteilen et al. [33]. Body temperature was maintained at 33°C–34°C using a heating pad during surgery. MIT‐001 (30 mg/kg, i.p.) was administered once or three times depending on the group allocation. Ferrostatin‐1 (1 mg/kg, i.p., Sigma‐Aldrich, St. Louis, MO, USA) was administered once or five times depending on the group allocation. Mice were sacrificed at specified time points—24 h after the final MIT‐001 administration. At the endpoint, blood and kidney tissues were harvested for analysis. Throughout the experiment, no animals showed signs of pain, distress, or morbidity. Euthanasia was performed humanely via ketamine anaesthesia followed by cervical dislocation to minimise suffering.

### 4.7 Blood and Tissue Preparation

Tissues were prepared as previously described [34]. Briefly, blood was collected from the inferior vena cava of anaesthetised mice. Blood samples were placed in microcentrifuge tubes (4°C). For blood urea nitrogen and s‐Cr assessment, serum was separated by centrifugation at 4°C for 10 min. Immediately after euthanasia, each animal's left kidney was excised and cut into three sections. Two pieces of kidney were flash‐frozen in liquid nitrogen and stored at −70°C for subsequent protein and RNA extraction analysis. The other kidney section was fixed in 4% paraformaldehyde at 4°C and then embedded in Paraplast (Sherwood Medical, St. Louis, MO, USA) for microscopy. Paraffinised sections were cut to a thickness of 4 μm.

### 4.8 Tissue Injury Score

Kidney sections were deparaffinised with xylene, stained with H&E and Masson trichrome, and examined under a microscope (Olympus BX51: Olympus, Tokyo, Japan). Six consecutive fields were examined at 200× magnification, and tissue injury scores were averaged per slide. For the H&E sections, renal cortical vacuolization, proximal tubule simplification, renal cortical vacuolization and peritubular/proximal tubule leukocyte infiltration were evaluated and scored as follows: normal, 0; < 25% injury, 1; > 25% to 50% injury, 2; > 50% to 75% injury, 3; and > 75% to 100% injury, 4. The injury scoring was performed by an experienced pathologist blinded to the experimental protocol of the specimen. Masson trichrome staining was used for measuring the inflammatory cell accumulation and collagen deposition in kidney tissue sections.

### 4.9 Western Blot Analysis

Proteins were extracted with buffer containing 1 M phosphate‐buffered saline, 5 M EDTA, and 0.5% Triton X‐100 or NP‐40 buffer (Invitrogen, Waltham, MA, USA) containing 1X phosphatase inhibitor cocktail and 1× protease inhibitor cocktail (Roche, Basel, Switzerland). After centrifugation (13,000 rpm for 10 min, 4°C), the supernatant was collected for Western blot analysis. Protein (20 μg/lane) was electrophoresed on 10%–15% SDS gel and transferred to polyvinylidene fluoride membranes and blocked with 5% nonfat dry milk for 1 h at room temperature. In vitro and in vivo experiments treated with TGF‐β, they were incubated overnight at 4°C with α‐tubulin, E‐cadherin and Snail (1:1000, 1:1000 and 1:1000 respectively: Cell Signalling Technology, Danvers, MA, USA), collagen IV, α‐SMA, Twist, HMGB1, *GPX4* and 4‐HNE (1:1000, 1:1000, 1:1000, 1:1000, 1:1000 and 1:1000 respectively: Abcam, Cambridge, UK), GAPDH, NF‐κB p65 and Nrf2 (1:1000, 1:1000 and 1:1000 respectively: Santa Cruz Biotechnology, Dallas, TX, USA), HO‐1 (1:1000, SPA‐895: Enzo Life Sciences, Vienna, Austria) and xCT (1:1000, PA1‐16893: Invitrogen, Waltham, MA, USA). The membranes were incubated with horseradish peroxidase–conjugated antirabbit immunoglobulin G secondary antibodies (1:2000: Abfrontier Co. Ltd., Seoul, ROK) and horseradish peroxidase–conjugated antimouse immunoglobulin G secondary antibodies (1:2000: Abfrontier Co. Ltd.) for 2 h at room temperature. In vitro experiments treated with RSL3, they were incubated overnight at 4°C with primary antibodies against GPX4 (59735S, Cell Signalling Technology), Nrf2 (33649S, Cell Signalling Technology) and β‐actin (A5441, Sigma). They were then incubated with antirabbit IgG, HRP‐linked antibody (7074S, Cell Signalling Technology), and Got anti‐Mouse IgG Secondary Antibody [HRP] (HAF007, R&D Systems) for 2 h at room temperature. Protein bands were visualised using a chemiluminescence detection kit (Thermo Fisher Scientific, South Logan, UT, USA). The same membranes were subsequently used for α‐tubulin immune detection, and equal protein loading was ensured. Optical density for quantification was obtained using the Gel‐Pro Analyser software application (version 3.1: Media Cybernetics, Silver Spring, MD, USA).

### 4.10 Immunohistochemistry and Histology Staining

Immunohistochemistry was performed by cutting kidney tissue into 4 μm sections, which were placed on glass slides, deparaffinised with xylene, and hydrated in a series of alcohol solutions. Peroxide blockade was performed using 3% H_2_O_2_ in methanol for 10 min at room temperature. Collagen IV, α‐SMA, 4‐HNE and TGF‐β (1:200, 1:200, 1:200 and 1:500 respectively: Abcam), E‐cadherin (1:200: Cell Signalling Technology) and F4/80 (1:200, MCA 497GA: Bio‐Rad [AbD Serotec], Oxford, UK) were incubated overnight at 4°C. After washing, secondary antibodies were added, and the samples were further incubated at room temperature for 30 min. The slides were subjected to DAB immunostaining using the REAL EnVision Detection System, Peroxidase/DAB+ and Rabbit/Mouse Kit (Dako, Carpinteria, CA, USA). The slides were then counterstained with Meyer's haematoxylin, dehydrated, and covered with a coverslip.

### 4.11 Terminal Deoxynucleotidyl Transferase dUTP Nick End Labelling Staining

Paraffin sections (4 μm) were deparaffinised and incubated in 3% H_2_O_2_ at room temperature to remove endogenous peroxidase activity. Sections were tested for TUNEL staining using the In Situ Cell Death Detection Kit, POD (11,684,817,910: Roche, Basel, Switzerland) according to the manufacturer's recommendations. TUNEL‐positive cells were identified as fluorescent signals using a fluorescence microscope. To evaluate apoptosis semiquantitatively, six microscopic fields were randomly selected at 200× magnification. The apoptosis index (TUNEL‐positive cell number/DAPI‐positive cells) was calculated using the Image Pro Plus 6.0 software application (Media Cybernetics).

### 4.12 Dihydroethidium Staining

Paraffin elongation sections (4 μm) were deparaffinised and incubated with a solution of dihydroethidium (D11347: Invitrogen) at 37°C for 30 min. They were coverslipped with Fluoroshield (Roche) containing DAPI and then observed under a fluorescence microscope.

### 4.13 RT‐PCR

In vitro and in vivo experiments treated with TGF‐β, total RNA was extracted using a Total RNA Miniprep Kit (T2010S: NEB, Ipswich, MA, USA). PCR was used to amplify these specific cDNAs: UCP‐1 (primer: sense 5′‐TCT CTC AGG ATC GGC CTC TA‐3′, antisense 5′‐GCC CAA TGA ATA CTG CCA CT‐3′), MnSOD (primer: sense 5′‐GGA AGC CAT CAA ACG TGA CT‐3′, antisense 5′‐CTG ATT TGG ACA AGC AGC AA‐3′), *GPX4* (primer: sense 5′‐CTT CCC GTG TAA CCA GTT CG‐3′, antisense 5′‐TCA CGC AGA TCT TGC TGA AC‐3′), MCP‐1 (primer: sense 5′‐CCC CAG TCA CCT GCT GTT AT‐3′, antisense 5′‐TCT CCT TGG CCA CAA TG‐3′), OPN (primer: sense 5′‐CTG GAT GAC CAG AGT GCT GA‐3′, antisense 5′‐TGA AAT TCA TGG CTG TGG AA‐3′), 4‐HNE (primer: sense 5′‐GGA TGG TAA CCA CCT GCT GT‐3′, antisense 5′‐TGC CAA AGA GAT TGT GCT TG‐3′) and GAPDH (primer: sense 5′‐GAG TCA ACG GAT TTG GTC GT‐3′, antisense 5′‐GAC AAG CTT CCC GTT CTC AG‐3′). In in vitro experiments treated with RSL3, total RNA was extracted using a RNeasy Mini Kit (Qiagen, Hilden, Germany). PCR was used to amplify these specific cDNAs: GPX4 (primer: sense 5′‐GTT TTC CGC CAA GGA CAT CG‐3′, antisense 5′‐ACT TCG GTC TTG CCT CAC TG‐3′), GAPDH (primer: sense 5′‐GCT CTC TGC TCC TCC TGT TC‐3′, antisense 5′‐CCA TGG TGT CTG AGC GAT GT‐3′). PCR was performed in an amplification reaction volume of 20 μL, consisting of 10 μL iQ SYBR Green PCR Master Mix (Qiagen Sciences Inc., Hilden, Germany), 2 μL primer, 2 μL cDNA and 6 μL H_2_O. Amplification and detection were performed using a thermal circulator (Rotor‐Gene 6000: Corbett Research Pty Ltd., Mortlake, NSW, Australia). These PCR conditions were used: denaturation at 95°C for 10 min, followed by 40 cycles of 10 s at 95°C, 15 s at annealing temperature (60°C for GAPDH, UCP‐1, MnSOD, *GPX4*, MCP‐1, OPN, 4‐HNE) and 20 s at 72°C. SYBR green fluorescence was measured at the end of each cycle using the comparative threshold cycle method. The results were evaluated using the ΔΔCt method.

### 4.14 Statistical Analysis

All data are expressed as means ± standard deviation. Multiple comparisons between groups, non‐parametric tests were performed using the Kruskal‐Wallis H test, followed by a post hoc Bonferroni correction. The IBM SPSS Statistics for Windows software application (version 20.0: IBM, Armonk, NY, USA) or Prism software (GraphPad, Boston, MA, USA) was used for the analysis. Differences between groups were considered significant at *p* < 0.05.

## Author Contributions

**Jin Ah Shin:** conceptualization (equal), data curation (equal), formal analysis (lead), investigation (equal), methodology (equal), writing – original draft (equal). **Yunkyeong Hwang:** conceptualization (equal), data curation (equal), funding acquisition (supporting), investigation (supporting), methodology (equal), writing – original draft (equal). **Hyerim Park:** data curation (supporting), methodology (supporting). **Hyunsu Choi:** data curation (supporting), methodology (supporting). **Yoon‐Kyung Chang:** investigation (supporting), resources (supporting), validation (supporting). **Ki Ryang Na:** resources (supporting), supervision (supporting). **Soon Ha Kim:** resources (supporting), validation (supporting). **Jae Young Kim:** methodology (supporting), resources (supporting). **Young Rok Ham:** conceptualization (equal), investigation (equal), project administration (equal), resources (equal), writing – review and editing (equal). **Dae Eun Choi:** conceptualization (equal), funding acquisition (lead), investigation (equal), resources (equal), supervision (lead), validation (lead), writing – review and editing (equal).

## Conflicts of Interest

The authors declare no conflicts of interest.

## Supporting information


**Figure S1:** Determining the optimal dose of MIT‐001 (A) At doses of 30 and 50 mg/kg, blood chemistry analysis showed significant reductions in blood urea nitrogen (BUN) and serum creatinine (s‐Cr), whereas the 10 mg/kg dose did not produce significant changes. (B) Representative kidney sections stained with haematoxylin and eosin (H&E) at Days 3 and 7 after renal IRI. Dilated renal tubules, tubular necrosis, and inflammatory cell infiltration indicated tubulointerstitial damage, while treatment with MIT‐001 at 30 and 50 mg/kg significantly improved the tubulointerstitial damage score. In contrast, the 10 mg/kg dose was not effective. WT, wild‐type mice, sham treatment (*n* = 5 for 3d, *n* = 5 for 7d); WT + M, wild‐type mice treated with MIT‐001 (*n* = 5 (10), *n* = 5 (30) and *n* = 5 (50) for 3d and *n* = 5 (10), *n* = 5 (30) and *n* = 5 (50) for 7d); IR 3d, untreated Day 3 renal IRI wild‐type mice (*n* = 5); IR 3d + M, Day 3 renal IRI mice treated with MIT‐001 (*n* = 5 (10), *n* = 5 (30) and *n* = 5 (50)); IR 7d, untreated Day 7 renal IRI mice (*n* = 5); IR 7d + M, Day 7 renal IRI mice treated with MIT‐001 (*n* = 5 (10), *n* = 5 (30) and *n* = 5 (50)); (10), 10 mg/kg of MIT‐001; (30), 30 mg/kg of MIT‐001; (50), 50 mg/kg of MIT‐001. (C) At 100 mg/kg, BUN and s‐Cr levels were reduced at Day 3 but did not reach statistical significance, and no improvement was observed at Day 7. (D) Representative H&E‐stained kidney sections at Day 3 after renal IRI. Treatment with MIT‐001 at 100 mg/kg significantly improved the tubulointerstitial damage score at Day 3; however, no statistically significant effect was observed at Day 7. WT, wild‐type mice, sham treatment (*n* = 5 for 3d and *n* = 5 for 7d); WT + M (100), wild‐type mice treated with MIT‐001 (*n* = 5 for 3d and *n* = 5 for 7d); IR 3d, untreated Day 3 renal IRI wild‐type mice (*n* = 5); IR 3d+ M (100), Day 3 renal IRI mice treated with MIT‐001 (*n* = 5); IR 7, untreated Day 7 renal IRI mice (*n* = 5); IR 7d+ M (100), Day 7 renal IRI mice treated with MIT‐001 (*n* = 5). (100), 100 mg/kg of MIT‐001; Original magnification, ×200. (Scale bar = 100 μm). Bar charts represent means ± standard deviation. Kruskal–Wallis *H* test, followed by a post hoc Bonferroni correction *p < 0.01, **p < 0.001.
**Figure S2:** The experimental protocols for the mice. (A) The WT + M and IR 3d + M group received a single dose of MIT‐001 (30 mg/kg ip) on second day, starting 24 h after IR or Sham operation. (B) The WT+ M and IR 7d + M group received three doses of MIT‐001 (30 mg/kg ip every second day) after IR or Sham operation. WT, wild‐type mice, sham treatment (*n* = 5 for 3d, *n* = 5 for 7d); WT + M, wild‐type mice treated with MIT‐001 (*n* = 7 for 3d, *n* = 7 for 7d); IR 3d, untreated Day 3 renal IRI wild‐type mice (*n* = 10); IR 3d + M, Day 3 renal IRI mice treated with MIT‐001 (*n* = 10); IR 7d, untreated Day 7 renal IRI mice (*n* = 10); IR 7d + M, Day 7 renal IRI mice treated with MIT‐001 (*n* = 10). (C) IR 3d + F group received a single dose of F (1 mg/kg ip) on second day, starting 24 h after IR. IR 7d + M group received a five dose of F (1 mg/kg ip) once daily from Day 2 to Day 6 h after IR. IR 3d, untreated Day 3 renal IRI wild‐type mice (*n* = 7); IR 3d + M, Day 3 renal IRI mice treated with MIT‐001 (*n* = 7); IR 3d + F, Day 3 renal IRI mice treated with ferrostatin‐1 (*n* = 7); IR 7d, untreated Day 7 renal IRI mice (*n* = 7); IR 7d + M, Day 7 renal IRI mice treated with MIT‐001 (*n* = 7); IR 7d + F, Day 7 renal IRI mice treated with ferrostatin‐1 (*n* = 7).

## Data Availability

The data supporting the findings of this study are available upon request from the corresponding author.
