# Supplementary material for: Renoprotective Effects of MIT‐001 in Ischemia–Reperfusion Injury: Modulation of Ferroptosis, ROS and Fibrotic Markers
Source: J Cell Mol Med. 2025 Oct 22;29(20):e70914. doi: 10.1111/jcmm.70914 (PMC12544698; doi:10.1111/jcmm.70914)
Supplement: Supplementary file 1 — Figure S1: Determining the optimal dose of MIT‐001 (A) At doses of 30 and 50 mg/kg, blood chemistry analysis showed significant reductions in blood urea nitrogen (BUN) and serum creatinine (s‐Cr), whereas the 10 mg/kg dose did not produce significant changes. (B) Representative kidney sections stained with haematoxylin and eosin (H&E) at Days 3 and 7 after renal IRI. Dilated renal tubules, tubular necrosis, and inflammatory cell infiltration indicated tubulointerstitial damage, while treatment with MIT‐001 at 30 and 50 mg/kg significantly improved the tubulointerstitial damage score. In contrast, the 10 mg/kg dose was not effective. WT, wild‐type mice, sham treatment (n = 5 for 3d, n = 5 for 7d); WT + M, wild‐type mice treated with MIT‐001 (n = 5 (10), n = 5 (30) and n = 5 (50) for 3d and n = 5 (10), n = 5 (30) and n = 5 (50) for 7d); IR 3d, untreated Day 3 renal IRI wild‐type mice (n = 5); IR 3d + M, Day 3 renal IRI mice treated with MIT‐001 (n = 5 (10), n = 5 (30) and n = 5 (50)); IR 7d, untreated Day 7 renal IRI mice (n = 5); IR 7d + M, Day 7 renal IRI mice treated with MIT‐001 (n = 5 (10), n = 5 (30) and n = 5 (50)); (10), 10 mg/kg of MIT‐001; (30), 30 mg/kg of MIT‐001; (50), 50 mg/kg of MIT‐001. (C) At 100 mg/kg, BUN and s‐Cr levels were reduced at Day 3 but did not reach statistical significance, and no improvement was observed at Day 7. (D) Representative H&E‐stained kidney sections at Day 3 after renal IRI. Treatment with MIT‐001 at 100 mg/kg significantly improved the tubulointerstitial damage score at Day 3; however, no statistically significant effect was observed at Day 7. WT, wild‐type mice, sham treatment (n = 5 for 3d and n = 5 for 7d); WT + M (100), wild‐type mice treated with MIT‐001 (n = 5 for 3d and n = 5 for 7d); IR 3d, untreated Day 3 renal IRI wild‐type mice (n = 5); IR 3d+ M (100), Day 3 renal IRI mice treated with MIT‐001 (n = 5); IR 7, untreated Day 7 renal IRI mice (n = 5); IR 7d+ M (100), Day 7 renal IRI mice treated with MIT‐001 (n = 5). [file JCMM-29-e70914-s001.docx]

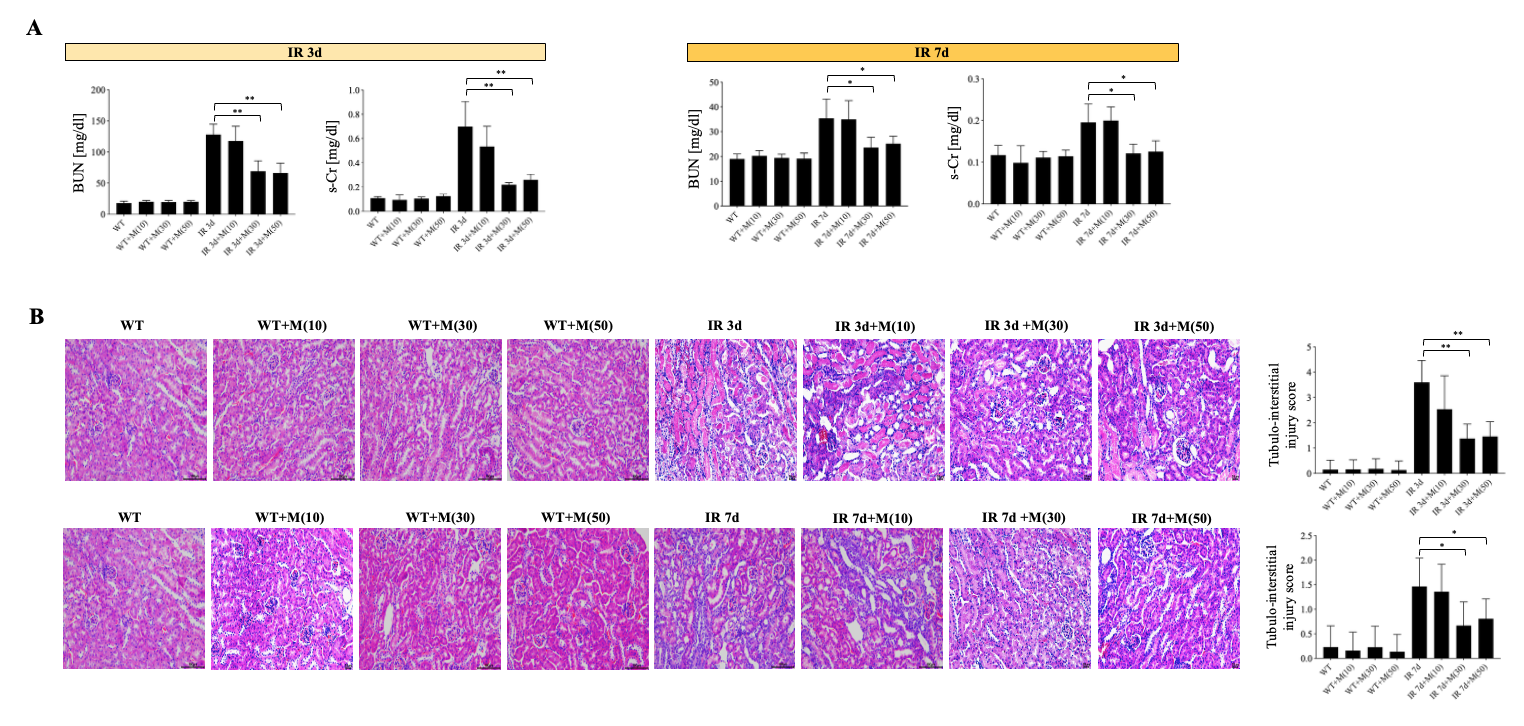

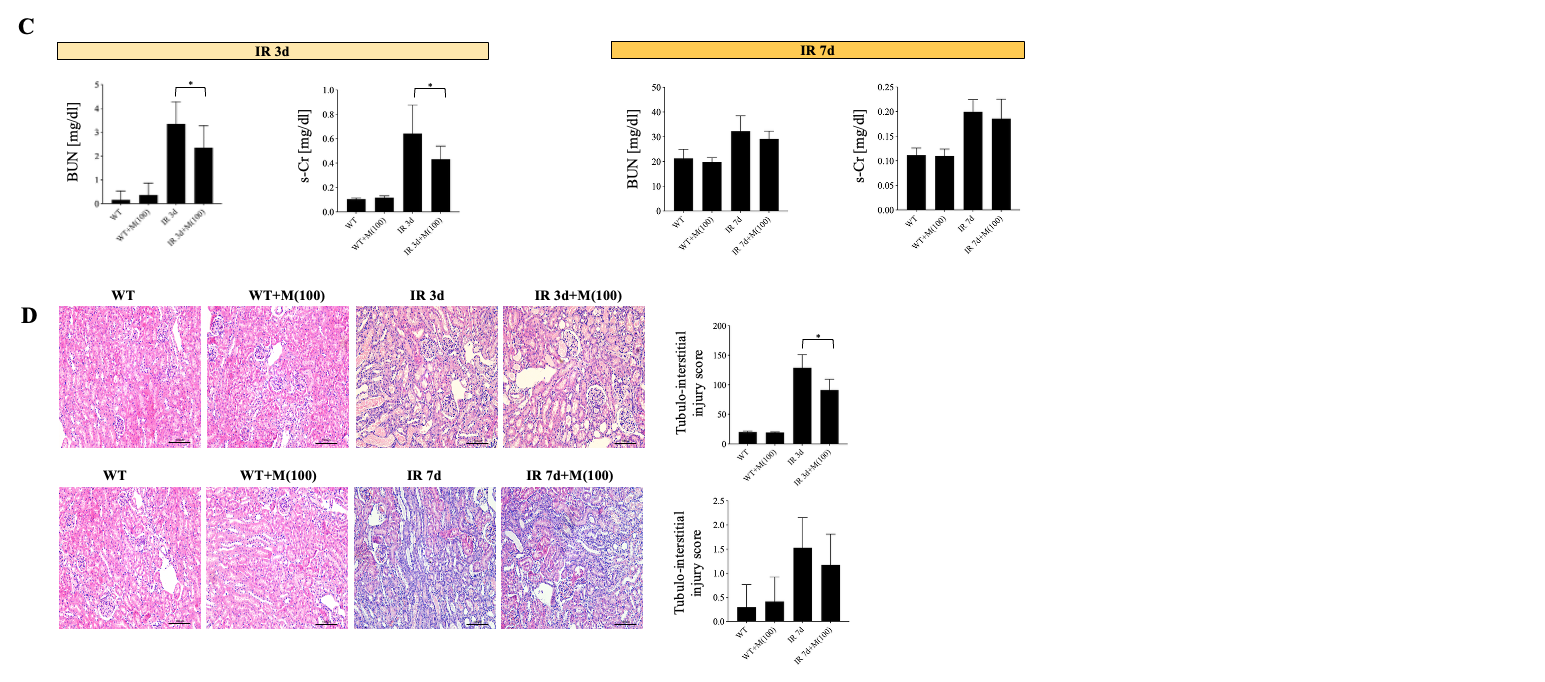


Supplementary Fig 1. Determining the optimal dose of MIT-001 (A) At doses of 30 and 50 mg/kg, blood chemistry analysis showed significant reductions in blood urea nitrogen (BUN) and serum creatinine (s-Cr), whereas the 10 mg/kg dose did not produce significant changes. (B) Representative kidney sections stained with hematoxylin and eosin (H&E) at days 3 and 7 after renal IRI. Dilated renal tubules, tubular necrosis, and inflammatory cell infiltration indicated tubulointerstitial damage, while treatment with MIT-001 at 30 and 50 mg/kg significantly improved the tubulointerstitial damage score. In contrast, the 10 mg/kg dose was not effective. WT, wild-type mice, sham treatment (*n*=5 for 3d, *n*=5 for 7d); WT + M, wild-type mice treated with MIT-001(*n*=5 (10), *n*=5 (30), and *n*=5 (50) for 3d and *n*=5 (10), *n*=5 (30), and *n*=5 (50) for 7d); IR 3d, untreated day 3 renal IRI wild-type mice (*n*=5); IR 3d + M, day 3 renal IRI mice treated with MIT-001(*n*=5 (10), *n*=5 (30), and *n*=5 (50)); IR 7d, untreated day 7 renal IRI mice(*n*=5); IR 7d + M, day 7 renal IRI mice treated with MIT-001(*n*=5 (10), *n*=5 (30), and *n*=5 (50)); (10), 10mg/kg of MIT-001; (30), 30mg/kg of MIT-001; (50), 50mg/kg of MIT-001. (C) At 100 mg/kg, BUN and s-Cr levels were reduced at day 3 but did not reach statistical significance, and no improvement was observed at day 7. (D) Representative H&E-stained kidney sections at day 3 after renal IRI. Treatment with MIT-001 at 100 mg/kg significantly improved the tubulointerstitial damage score at day 3; however, no statistically significant effect was observed at day 7. WT, wild-type mice, sham treatment (*n*=5 for 3d and *n*=5 for 7d); WT + M(100), wild-type mice treated with MIT-001(*n*=5 for 3d and *n*=5 for 7d); IR 3d, untreated day 3 renal IRI wild-type mice (*n*=5); IR 3d + M(100), day 3 renal IRI mice treated with MIT-001(*n*=5); IR 7d, untreated day 7 renal IRI mice(*n*=5); IR 7d + M(100), day 7 renal IRI mice treated with MIT-001(*n*=5). (100), 100mg/kg of MIT-001;Original magnification, ×200. (Scale bar = 100 μm)**.** Bar charts represent means ± standard deviation. Kruskal-Wallis H test, followed by a post hoc Bonferroni correction *p < 0.01. **p < 0.001.


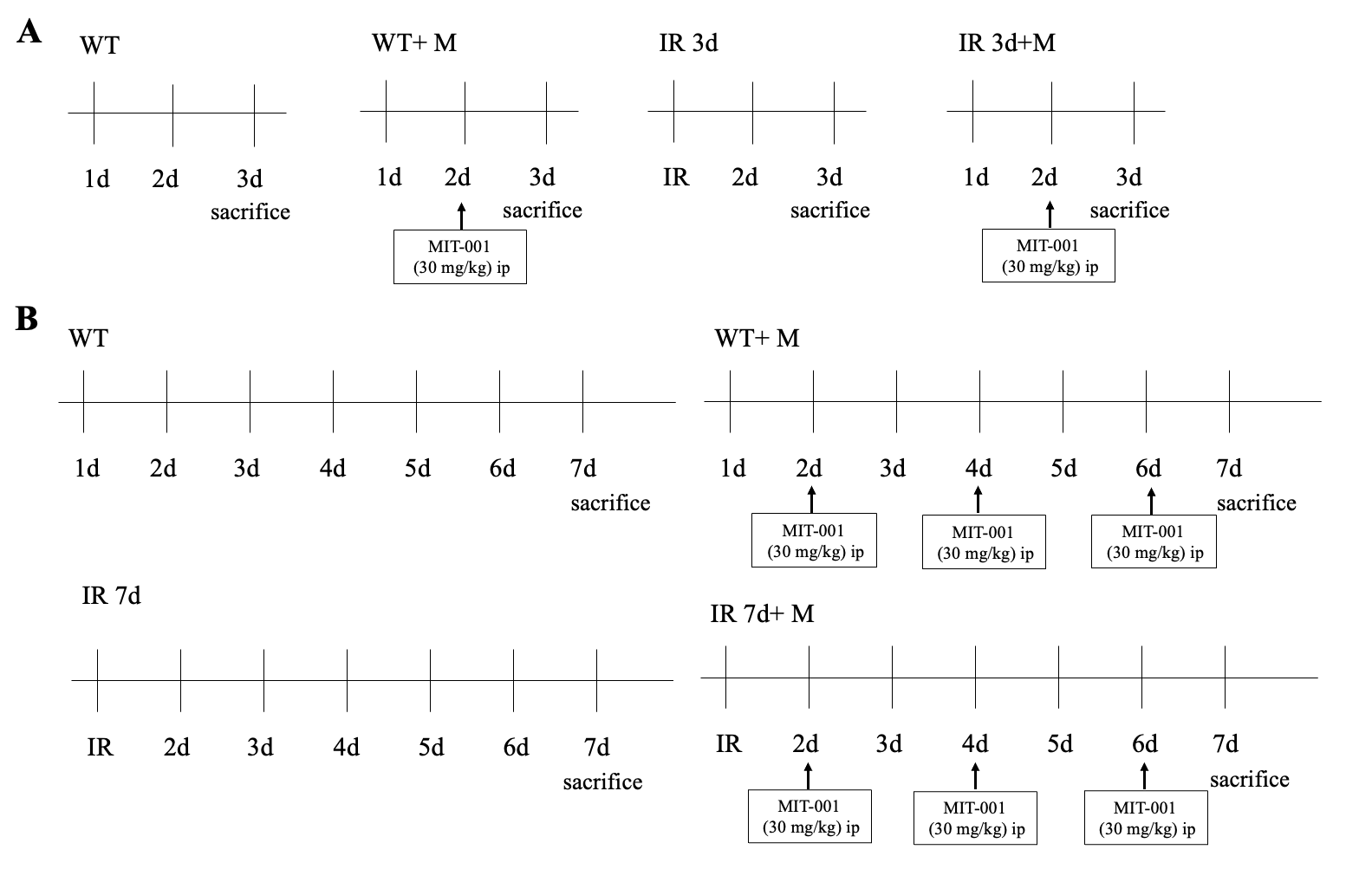


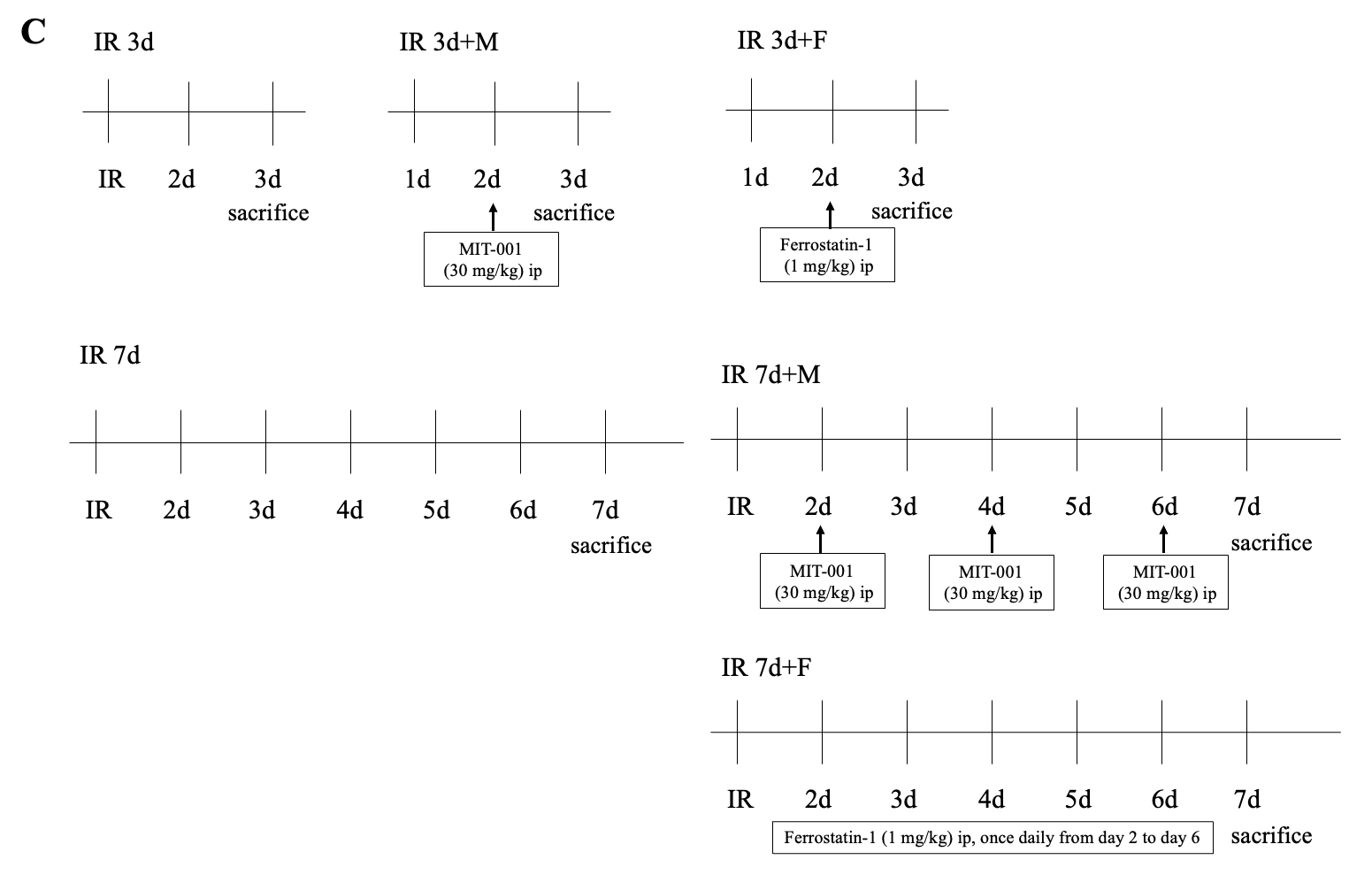


Supplementary Fig 2. The experimental protocols for the mice. (A) The WT+M and IR 3d + M group received a single dose of MIT-001 (30 mg/kg ip) on second day, starting 24 h after IR or Sham operation. (B) The WT+ M and IR 7d + M group received three doses of MIT-001 (30 mg/kg ip every second day) after IR or Sham operation. WT, wild-type mice, sham treatment (*n*=5 for 3d, *n*=5 for 7d); WT + M, wild-type mice treated with MIT-001(*n*=7 for 3d, *n*=7 for 7d); IR 3d, untreated day 3 renal IRI wild-type mice(*n*=10); IR 3d + M, day 3 renal IRI mice treated with MIT-001(*n*=10); IR 7d, untreated day 7 renal IRI mice(*n*=10); IR 7d + M, day 7 renal IRI mice treated with MIT-001(*n*=10). (C) IR 3d + F group received a single dose of F (1 mg/kg ip) on second day, starting 24 h after IR. IR 7d + M group received a five dose of F (1 mg/kg ip) once daily from day 2 to day 6h after IR. IR 3d, untreated day 3 renal IRI wild-type mice (*n*=7); IR 3d + M, day 3 renal IRI mice treated with MIT-001(*n*=7); IR 3d + F, day 3 renal IRI mice treated with ferrostatin-1(*n*=7); IR 7d, untreated day 7 renal IRI mice(*n*=7); IR 7d + M, day 7 renal IRI mice treated with MIT-001(*n*=7); IR 7d + F, day 7 renal IRI mice treated with ferrostatin-1 (*n*=7).
